# OCT Angiography Assessment of Type 1 Diabetes Mellitus Patients Without Diabetic Retinopathy: A 3-Year Follow-Up Study

**DOI:** 10.3390/diagnostics15131703

**Published:** 2025-07-03

**Authors:** Alexandra Oltea Dan, Carmen Luminița Mocanu, Alin Ștefan Ștefănescu-Dima, Andreea Cornelia Tănasie, Veronica Elena Maria, Anca Elena Târtea, Andrei Theodor Bălășoiu

**Affiliations:** 1 Department of Ophthalmology, University of Medicine and Pharmacy of Craiova, 200349 Craiova, Romania; puiu.alexandra.oltea@gmail.com (A.O.D.); carmen.mocanu@umfcv.ro (C.L.M.); andrei.balasoiu@umfcv.ro (A.T.B.); 2 Department of Physiology, University of Medicine and Pharmacy of Craiova, 200349 Craiova, Romania; 3 Department of Pediatrics, University of Medicine and Pharmacy of Craiova, 200349 Craiova, Romania; veronica.maria@umfcv.ro; 4 Department of Neurology, University of Medicine and Pharmacy of Craiova, 200349 Craiova, Romania; anca.tartea@umfcv.ro

**Keywords:** OCT angiography, retina, type 1 diabetes mellitus

## Abstract

**Background/Objectives:** This study aims to investigate the progression of retinal microvascular changes using OCTA in young T1DM patients without clinical signs of DR over a period of 3 years. **Methods**: This prospective, longitudinal study analyzed OCT angiograms of T1DM patients without clinical signs of DR. It included 40 T1DM patients aged between 7 and 20 years old who formed the T1DM study group and 40 healthy subjects with similar demographic characteristics to the control group. The patients underwent comprehensive ophthalmic examination and OCT imaging using a Retina Wide protocol (6 × 6 mm). We analyzed the following microvascular retinal parameters: FAZ area, perimeter and circularity and superficial capillary plexus (SCP) and deep capillary plexus (DCP) vessel density. **Results**: Statistically significant differences between the two groups were identified for the following parameters: the mean FAZ area at follow-up (0.38 ± 0.13) was larger than the mean FAZ area at baseline (0.31 ±0.11), the mean FAZ perimeter at follow-up (3.22 ± 0.75) was larger than the mean FAZ perimeter at baseline (2.61 ± 00.52) and the men FAZ circularity index at follow-up (0.47 ± 0.13) was decreased compared to the FAZ circularity index at baseline (0.56 ± 0.12). A statistically significant difference were also registered for the following parameter: the total SCP and DCP vessel density was decreased at follow-up (37.47 ± 1.57) compared to baseline (38.79 ± 1.00). **Conclusions**: OCTA long-term monitoring of T1DM patients represents an effective method for tracking progressive changes in FAZ parameters and capillary plexus vascular density.

## 1. Introduction

Type 1 diabetes mellitus (T1DM) is a chronic autoimmune condition characterized by the immune-mediated destruction of insulin-producing pancreatic β-cells, leading to absolute insulin deficiency [1]. The global incidence and prevalence of T1DM continues to rise yearly [2], particularly among children and adolescents [3]. Recent estimates indicate that over 9 million individuals are affected worldwide, with an incidence of 15 per 100,000 people and a prevalence of 9.5% [4]. Chronic hyperglycemia in T1DM contributes to the development of long-term microvascular complications [5], including diabetic retinopathy (DR), which remains a leading cause of vision impairment in working-age populations [6].

Currently, approximately 285 million individuals globally are diagnosed with DR, highlighting the urgent need for effective screening and management strategies [7]. Although DR is clinically diagnosed based on fundoscopic findings—such as microaneurysms, hemorrhages, exudates and neovascularization—the retinal neurovascular dysfunction begins long before the visible signs appear on fundus examination [8]. These subtle alterations include capillary nonperfusion, early disruption of the blood–retina barrier and neurodegeneration [9], suggesting a preclinical phase of DR that currently remains undetectable through conventional screening methods.

For decades, imaging devices have represented an indispensable tool in ophthalmology diagnosis, providing high-resolution imaging critical for early diagnosis, disease monitoring and treatment guidance [10]. Optical coherence tomography angiography (OCTA) represents one of the most recent imaging techniques; it enables an in-depth visualization of the retinal and choroidal microvasculature without the need for contrast dye injection [11]. Unlike traditional techniques such as fluorescein angiography, OCTA does not require contrast agents, enabling detailed assessment of both the superficial and deep capillary plexuses [12]. This facilitates early detection of microvascular alterations associated with DR progression. Moreover, while conventional DR screening methods rely on manual interpretation, which is time-consuming and subjective, AI-assisted OCTA could enable rapid and objective image analysis, enhancing screening efficiency and reducing diagnostic delays [13].

OCTA retinal scans are based on capturing blood flow using repeated B-scans, offering three-dimensional information on microvascular morphology and perfusion of retinal layers [14]. OCTA assessment of key vascular parameters, such as the foveal avascular zone (FAZ) and the vascular density of the capillary networks, has revealed alterations in diabetic patients, even in the absence of clinically apparent DR [15]. The progression rate of DR varies significantly among patients, highlighting the importance of identifying specific biomarkers associated with the risk of progression of the disease. As retinal changes can also regress, it becomes essential to identify these biomarkers and the risk profiles of the patients in order to prevent or delay the clinical diagnosis of DR [16]. The OCTA imaging technique has proven its reliability in detecting the retinal microvascular changes associated with DR, even before the specific clinical signs become obvious. In their recent study, Cutruzzolà A et al. [17] highlighted that OCTA FAZ parameters and vessel density can be considered biomarkers for early microvascular alterations in adult T1DM patients without DR, concluding that further longitudinal studies are needed to clarify specific changes of these biomarkers in the development of DR. As most recent studies are cross-sectional and focus primarily on adult populations or patients with existing signs of DR, the novelty of the present study is that it uniquely focuses on a pediatric and adolescent T1DM population without clinical signs of DR using a prospective longitudinal design over three years. To our knowledge, few studies have tracked microvascular changes over time in this young demographic, making this work valuable for understanding the earliest retinal alterations in T1DM and informing strategies for timely intervention.

This study aims to investigate the progression of retinal microvascular changes using OCTA in young T1DM patients without clinical signs of DR over a period of 3 years. By identifying early biomarkers of retinal dysfunction, we aim to contribute to a better understanding of the microvascular changes that precede the clinical diagnosis of DR.

## 2. Materials and Methods

### 2.1. Patients

This prospective, longitudinal study analyzed OCT angiograms of T1DM patients without clinical signs of DR over a period of 3 years. The study population was selected from patients attending the ATB Ophthalmology Clinic, Craiova, Romania. It included 40 T1DM patients aged between 7 and 20 years old who formed the T1DM study group and 40 healthy subjects with similar demographic characteristics for the control group. The study protocol was approved by the Ethics Committee of the University of Medicine and Pharmacy Craiova (Project Identification Code 8612/07/06/2021) and was carried out in accordance with the rules of the Declaration of Helsinki, revised in 2013. Written informed consent was obtained from all the participating patients who met the inclusion criteria or their legal guardians if the patients were younger than 18 years old.

Our initial clinical study started in 2022, including 61 T1DM patients and 58 individuals for the control group. Baseline examinations were registered for all the study patients and statistically significant differences between the two groups were identified for the FAZ area, perimeter and circularity, as well as the vessel density of the SCP. These published results [18] suggested early microvascular dysfunction in T1DM patients compared to healthy subjects: the FAZ area and perimeter were statistically larger for the T1DM group, while the circularity index and the vascular density of the SCP were lower in the T1DM group compared to the control group. For the current research, we expanded on these findings by longitudinally tracking a subset of 40 patients from the initial T1DM group of 61 patients, as 19 patients were lost for follow-up and 2 patients developed clinical signs of non-proliferative DR.

The study patients met the following inclusion criteria for the T1DM group:

1. Patients diagnosed with T1DM, with duration of the disease of at least 5 years;
2. No clinical signs of DR and no other ophthalmic or systemic disorders, except for small refractive errors no higher than 2.00 diopters.

The exclusion criteria were

1. Patients with insufficient OCTA image quality (quality score < 7);
2. Patients with history of retinopathy of prematurity, vitreoretinal diseases including DR, and optic nerve disorders, as well as history of uveitis, ocular trauma, previous ocular surgery and ocular media opacities including cataracts or any other ocular or systemic conditions that may affect ocular system.

The control group consisted of individuals who visited the ophthalmology clinic for routine eye examinations and agreed to participate in the study. They were age-matched and had no ocular or systemic conditions, except for spherical or cylindrical refractive errors not exceeding 2.00 diopters.

For each participant, demographic and clinical characteristics were collected at the baseline visit, including age, duration of T1DM and glycemic control via HbA1c levels. The HbA1c average values were obtained by calculating the mean value of 4 measurements per year. All participants underwent full ophthalmic examination and OCTA imaging at baseline and at the 3-year follow-up visit.

### 2.2. Ophthalmic Examination

The ophthalmic examination for the study patients included best-corrected visual acuity assessment, refractive measurements with a Nidek Ark-1 refractor device, intraocular pressure using an I Care tonometer, biomicroscopic examination and dilated fundus examination using tropicamide 1% eye drops.

The OCTA retinal scans were obtained with the RevoNX 130 OCTA device (Optopol) using a Retina Wide 6 × 6 mm protocol. With the help of the OCTA software version 10.0.1, developed by OPTOPOL Technology, we analyzed the following microvascular retinal parameters: FAZ area, perimeter and circularity, and superficial capillary plexus (SCP) and deep capillary plexus (DCP) vessel density. The OCTA software segments the structural data to identify the boundaries of the SCP (located between the inner limiting membrane to the inner plexiform layer) and DCP (located between the inner nuclear layer and the outer plexiform layer). The vessel density is calculated as the percentage of vessel area with blood flow over the total area measured. The OCTA scans were captured at the same time of the day (in the morning) to rule out any diurnal variation in the OCTA metrics, as previous research has demonstrated that diurnal variation can influence OCTA metrics [19].

Poor-quality images with blink or motion artifacts were excluded from the analysis. The OCTA scans were captured at baseline and 3 years later for each patient and the progression tool of the OCTA software was used to evaluate the retinal changes.

### 2.3. Statistical Analysis

The data obtained from the OCTA reports was processed using Microsoft Excel 2021 (San Francisco, CA, USA). Statistical analysis of the results was then conducted to evaluate key parameters using unpaired 2-tailed *t*-tests to compare the data sets for the T1DM patients, with the significance value set at *p* < 0.05. For comparison of the TDM baseline, follow-up and control group, an ordinary one-way Anova test was conducted, with the significance value set at *p* < 0.05. The statistical analysis was conducted using the commercially available GraphPad Prism 10 software.

## 3. Results

The study included 80 participants aged between 7 and 20 years old; the mean age was 14.9 ± 3.57 years. The sample was divided into 40 patients with T1DM and 40 control subjects. The T1DM patients had a duration of disease between 5 years and 12 years, with a mean duration of 7.17 ± 2.08 years and average HbA1c values of 8.05 ± 1.42, as detailed in Table 1.

The OCTA parameters analyzed for the study included FAZ area (mm^2^), perimeter (mm) and circularity index (with the value 1 considered to be a perfect circle) and vessel density of the SCP and DCP. We analyzed these OCTA metrics for each patient at baseline and at the 3-year follow-up, as presented in Table 2.

**Figure 1:**
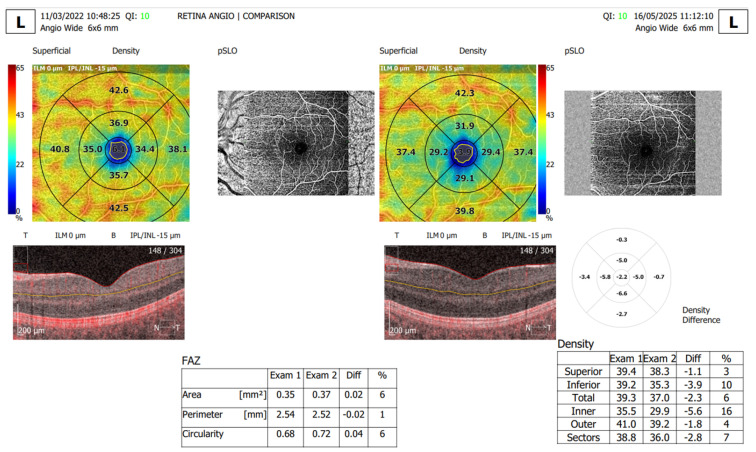
T1DM patients. Representative OCTA comparison report between the baseline visit and the follow-up visit 3 years later, showing alteration of the FAZ area and perimeter and the decrease in vessel density of the SCP.

**Figure 2:**
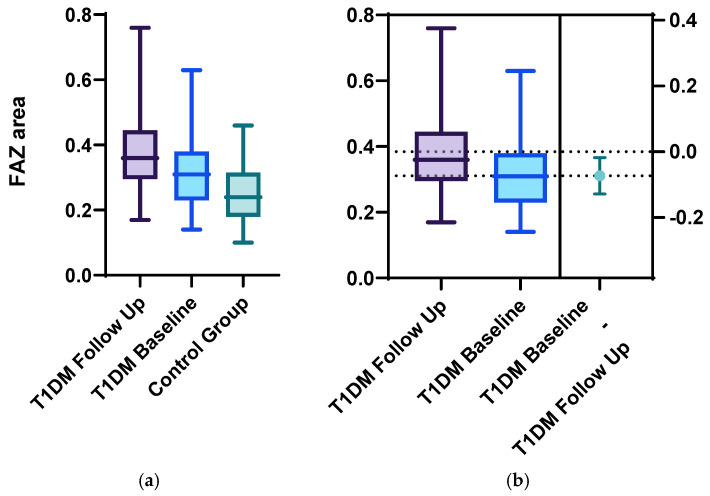
FAZ area at baseline and follow-up for the T1DM group compared with the control group using (**a**) ordinary one-way Anova analysis, with *p* < 0.01, and (**b**) unpaired 2-tailed *t*-test analysis of the follow-up and baseline FAZ area results, with *p* = 0.01.

**Figure 3:**
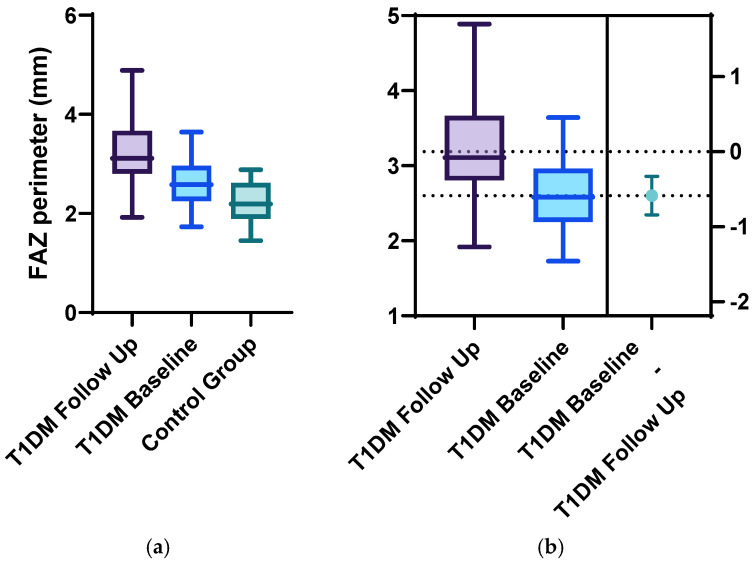
FAZ perimeter at baseline and follow-up for the T1DM group compared with the control group using (**a**) ordinary one-way Anova analysis, with *p* = 0.02, and (**b**) unpaired 2-tailed *t*-test analysis of the follow-up and baseline FAZ perimeter for the T1DM group, with *p* = 0.01.

**Figure 4:**
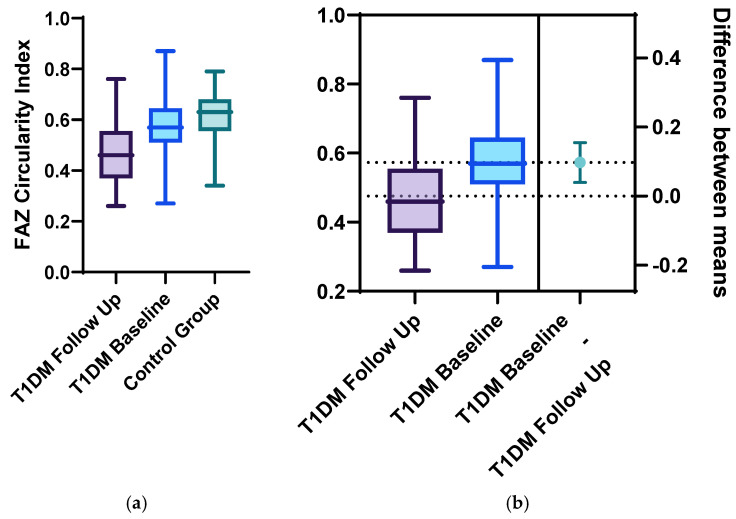
FAZ circularity index at follow-up and baseline for the T1DM group compared with the control group using (**a**) ordinary one-way Anova analysis, with *p* < 0.01, and (**b**) unpaired 2-tailed *t*-test analysis of the follow-up and baseline FAZ perimeter for the T1DM group, with *p* = 0.01.

Statistically significant differences between the two groups were identified for the following parameters: the mean FAZ area at follow-up (0.38 ± 0.13) was larger than the mean FAZ area at baseline (0.31 ± 0.11), the mean FAZ perimeter at follow-up (3.22 ± 0.75) was larger than the mean FAZ perimeter at baseline (2.61 ± 00.52) and the men FAZ circularity index at follow-up (0.47 ± 0.13) was decreased compared to the FAZ circularity index at baseline (0.56 ± 0.12) (Figure 1, Figure 2, Figure 3 and Figure 4).

**Figure 5:**
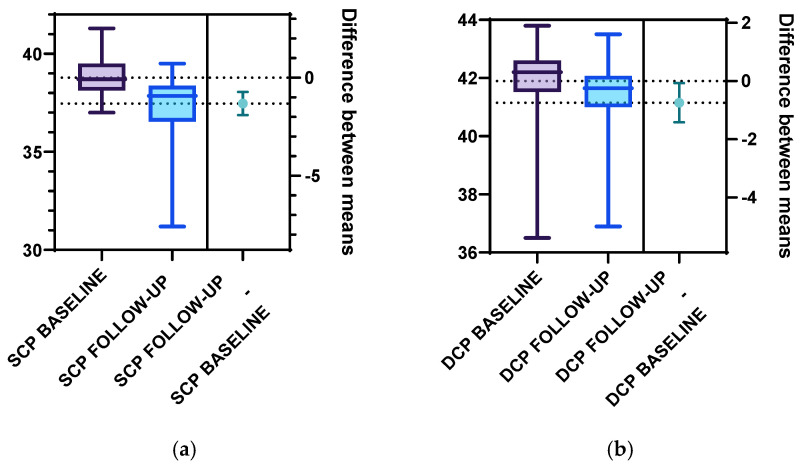
(**a**) SCP vessel density at baseline and follow-up for the T1DM group using unpaired 2-tailed *t*-test analysis, with *p* = 0.001. (**b**) DCP vessel density at baseline and follow-up for the T1DM using unpaired 2-tailed *t*-test analysis, with *p* = 0.03.

**Figure 6:**
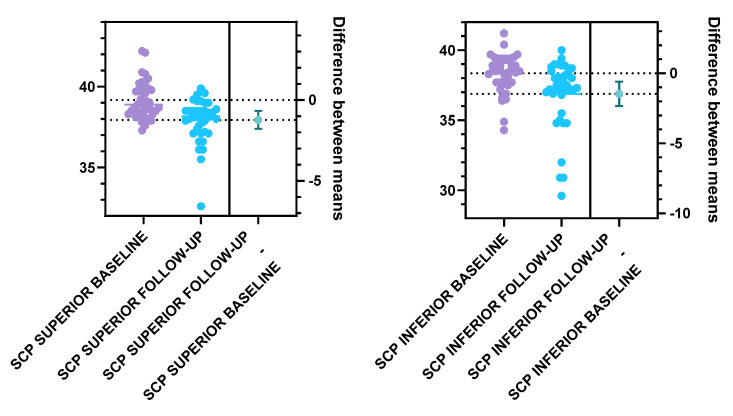
SCP vessel density in the superior and inferior parafoveal regions at baseline and follow-up, unpaired 2-tail *t*-test, *p* < 0.01.

Furthermore, we analyzed the vascular density of the SCP and DCP for the T1DM patients at baseline and follow-up (Figure 5, Table 2). Statistically significant differences were registered for the following parameters: The total SCP vessel density was decreased at follow-up (37.47 ± 1.57) compared to baseline (38.79 ± 1.00). Both superior and inferior parafoveal regions of the SCP had significantly lower vascular density at follow-up compared to baseline (Figure 6, Table 2). Regarding the DCP, the total vessel density was significantly lower at follow-up (41.15 ± 1.60) compared to baseline (41.90 ± 1.41), based only on the inferior DCP parafoveal sector (Table 2). We found no statistical difference between the follow-up and baseline analysis of the superior parafoveal region of the DCP.

## 4. Discussion

The results of our current study provide insights into the retinal microvascular alterations preceding DR using analysis of OCTA FAZ parameters and the vessel density of the capillary plexus over a three-year follow-up in T1DM patients without DR. Recent studies have provided both qualitative and quantitative OCTA data in diabetic patients, reporting a gradual decline in capillary density and branching complexity, along with a progressive increase in the average vascular caliber across various stages of DR [20].

Previous research on the OCTA metrics for monitoring the severity and progression of DR over a period of 3 years in adults with type 2 diabetes mellitus [21] indicated that progressive capillary loss serves as a valuable tool for monitoring the progression of DR. Our study specifically investigated young T1DM patients without clinical signs of DR, demonstrating that alterations in FAZ parameters and capillary vessel density are progressively worse on 3-year follow-up.

One of the earliest detectable OCTA changes preceding the clinical diagnosis of DR is reduced vascular density in the parafoveal region [22], while capillary non-perfusion is one of the characteristic OCTA manifestations of DR [23,24]. Our previous research, conducted on T1DM patients without DR, demonstrated alterations in FAZ parameters and SCP vessel density, suggesting early microvascular dysfunction compared to healthy subjects. We have built upon these findings by continuing to track some of these patients over time, assessing the progression of their OCTA retinal parameters to gain further insight into the dynamics of the microvascular changes.

Our findings are consistent with previous longitudinal studies on patients with clinical DR, which have demonstrated a significant decrease in parafoveal vessel density in the SCP alongside a reduction in the DCP that did not reach statistical significance over a period of 2 years [25]. Moreover, our study extends this evidence to a younger T1DM cohort that did not have clinical signs of DR, highlighting the potential of OCTA-derived metrics as sensitive, non-invasive biomarkers for early detection and progression monitoring of the retinal microvascular alterations preceding DR.

In our cohort of young T1DM patients without clinical DR, we observed a statistically significant increase in FAZ area and perimeter, along with a reduction in circularity over a 3-year period. These results imply that microvascular remodeling in the foveal region precedes visible signs of DR and support the idea that OCTA can detect subclinical retinal alterations. This underscores the potential role of FAZ parameters as early biomarkers for identifying patients at risk of developing DR, even before fundoscopic signs appear.

Our results demonstrate that FAZ parameters including area, perimeter, and circularity exhibit progressive alterations in T1DM patients with a disease duration of more than 5 years, reflecting early microvascular impairment and offering potential biomarkers for the onset and progression of DR.

Ensuring a rigorous quality check of OCTA vessel metrics is essential when comparing multiple examinations of the same patient in longitudinal studies and assessing disease progression. In our study, the examinations were conducted by experienced technicians and only high-quality images, without blink artefacts, were analyzed.

A limitation of the current research is represented by the small number of patients and the low variability of disease severity. At baseline, 61 patients were included in the T1DM group, but as they turned 18 years old, a portion of them were lost to follow-up, as they moved away for work or study purposes; moreover, 2 of them developed clinical signs of DR. The incidence of DR in the pediatric population shows that children are currently at low risk of developing the disease; however, the risk increases as they reach adolescence, with diabetic macular oedema or even proliferative DR being reported in this age group [26]. Considering that the risk of developing DR increases with the duration of T1DM, it is essential to ensure the best achievable glycemic control, as this is considered to be the most important preventive measure to delay retinal changes [27].

The results of the current study confirm that alterations in FAZ parameters and vessel density become more pronounced in T1DM patients as the duration of the disease increases, even if clinical DR is not visible on conventional fundus examination. Also, previous research conducted by Marwa Abdelshafy [28] demonstrated that the FAZ area and perimeter increase with disease duration in patients with clinically diagnosed DR. Multiple studies have demonstrated the high degree of repeatability and reliability of OCTA in measuring vascular changes in patients with DR compared to healthy subjects, associating these vascular changes with several degrees of DR severity [29,30]. Moreover, a recent meta-analysis also found significant FAZ alterations in prediabetic individuals compared to healthy controls, suggesting that such changes may occur even prior to the clinical onset of diabetes [31]. This further supports the utility of OCTA in identifying subclinical retinal microvascular damage and reinforces its role in early screening and disease monitoring.

In ophthalmic imaging, artificial intelligence (AI) has become integral in supporting clinical practice through the application of machine learning (ML) and deep learning (DL) technologies [32]. DL represents a specialized branch of ML that uses a complex architecture of neuron-like nodes that mimic the structure of the human brain, giving rise to the term “Convolutional Neural Networks” (CNNs) for systems that specialize in image recognition and classification [13]. DR is one of the primary causes of vision impairment worldwide, highlighting the importance of early automated detection for effective management. Automated DR classification has recently benefited from significant progress through the use of deep learning models, with CNNs and Vision Transformer (ViT) architectures being the most widely adopted due to their ability to capture spatial hierarchies and local patterns in images [33]. Although CNNs and ViT have demonstrated impressive performance in classifying the severity of DR, imaging biomarkers that precede the clinical signs of DR need further study.

The current AI applications in DR primarily focus on identifying the classical signs of DR such as hemorrhages, microaneurysms, exudates and macular edema. However, through our research, we aim to contribute to the development of guidelines for predicting progression towards DR. Moreover, based on the ocular data, AI algorithms could help predict the systemic impact of diabetes mellitus [34]. Considering that the OCTA technique enables direct, noninvasive in vivo visualization of the retinal microvascular alterations, it is reasonable to consider that similar microvascular changes may occur elsewhere in the body, such as in the heart or kidneys [35,36]. This highlights the relevance of OCTA retinal parameters as potential markers for systemic microvascular health in diabetic patients.

## 5. Conclusions

In conclusion, our findings highlight a significant increase in the FAZ area and perimeter, with a decrease in FAZ circularity, as well as a decrease in the vascular density of the SCP and DCP in the parafoveal region in T1DM patients over a 3-year follow-up period. Long-term OCTA monitoring of T1DM patients represents an effective method for tracking the progressive changes in FAZ parameters and the capillary plexus vascular density. By providing high-resolution imaging, the OCTA retinal evaluation of these patients enables detection of retinal microvascular abnormalities, which may lead to personalized management strategies for improved glycemic control.

## Figures and Tables

**Table 1:** Clinical characteristics of the study participants.

Clinical Characteristics	T1DM Group	Control Group	*p* Value
Gender (male/female)	29/11	17/23	-
Age (years)	14.9 ± 3.57	15.5 ± 2.75	0.39
T1DM duration	7.17 ± 2.08	-	-
HbA1c (mean %)	8.05 ± 1.42	-	-

**Table 2:** Analysis of OCTA parameters of the T1DM patients at follow-up and baseline using a unpaired two-tailed *t*-test, with * *p* < 0.05 considered statistically significant.

OCTA Parameters	T1DM Follow-Up	T1DM Baseline	*p* Value
FAZ area (mm^2^)	0.38 ± 0.13	0.31 ± 0.11	0.01 *
FAZ Perimeter (mm)	3.22 ± 0.75	2.61 ± 00.52	<0.01 *
FAZ circularity	0.47 ± 0.13	0.56 ± 0.12	<0.01 *
SCP total vessel density (%)	37.47 ± 1.57	38.79 ± 1.00	<0.01 *
SCP superior vessel density (%)	37.95 ± 1.33	39.18 ± 1.14	<0.01 *
SCP inferior vessel density (%)	36.89 ± 2.40	38.35 ± 1.35	<0.01 *
DCP total vessel density (%)	41.15 ± 1.60	41.90 ± 1.41	0.03 *
DCP superior vessel density (%)	41.51 ± 1.37	42.03 ± 1.64	0.12
DCP inferior vessel density (%)	40.78 ± 2.26	41.63 ± 1.46	0.02 *

## Data Availability

The authors declare that the data included in the study is available from the corresponding authors upon reasonable request.

## Abbreviations

The following abbreviations are used in this manuscript:

T1DM	Type 1 Diabetes Mellitus
DR	Diabetic Retinopathy
OCTA	Optical Coherence Tomography Angiography
FAZ	Foveal Avascular Zone
SCP	Superficial Capillary Plexus
DCP	Deep Capillary Plexus
AI	Artificial Intelligence
ML	Machine Learning
DL	Deep Learning
CNNs	Convolutional Neural Networks
ViT	Vision Transformer
